# Editorial: The musical brain, volume II

**DOI:** 10.3389/fnins.2024.1424961

**Published:** 2024-07-29

**Authors:** Jonathan Fritz, Amy Belfi, Jessica Grahn, John Iversen, Isabelle Peretz, Robert Zatorre

**Affiliations:** ^1^Center for Neural Science, New York University, New York, NY, United States; ^2^Department of Psychological Science, Missouri University of Science and Technology, Rolla, MO, United States; ^3^Department of Psychology, Brain and Mind Institute, Western University, London, ON, Canada; ^4^Department of Psychology, Neuroscience and Behavior, Swartz Center for Computational Neuroscience, McMaster University, Hamilton, ON, Canada; ^5^Department of Psychology, Université de Montréal, Montreal, QC, Canada; ^6^Department of Psychology, McGill University, Montreal, QC, Canada

**Keywords:** music, pitch, brain, language, auditory, reward, perception

Music exists in all human cultures, and plays an important role throughout our lives. The interactions between music and our brain are complex, engaging multiple neural circuits and networks related to sensory perception and prediction, attention, learning and memory, emotion, aesthetics and reward, and expert motor skills for performing vocal and instrumental music. Neuroscience research is actively engaged in exploring how the human brain “hears” music, identifying the neural mechanisms underlying the perception of rhythm, pitch, timbre, harmony and melody, and the development of musical awareness, expression and creativity. Experiments in non-human species are making important contributions to our understanding of some of the precursor components of musicality, and their underlying neural mechanisms, with implications for the evolution of musicality. In this Research Topic (*Musical Brain II*), we have sought to gather a broad range of topics in neuroscience and psychoacoustics covering the neural basis for perception of music and aspects of sound related to music.

There has been a steadily growing interest in the representation of music in the brain over the past decade since the first Frontiers Research Topic on the *Musical Brain I* (2013) was published. The articles in *Musical Brain I* covered many topics including the neural basis of music perception in the general context of human sound perception, neuroplasticity, musical awareness before birth, music development in children, adolescents and adults, musical memory, comparative studies of animal auditory processing, auditory perception and cognition. There have been over 600,000 views of these articles in the past 10 years. The most viewed article (half of the 600,000 views) was by Gervain et al., entitled “*Valproate reopens critical period learning of absolute pitch*” that demonstrated a statistical increase in perfect pitch in adults who had been given valproate.

This article, like many of the others in *Musical Brain I*, stimulated subsequent research that suggests that absolute pitch may also be acquired in adulthood without valproate by intensive gamified, computerized training (Wong et al., 2020). Further work on the topic of absolute pitch is included in the current Research Topic (Leite Filho et al.).

In the earlier Musical Brain I Special Issue, there was also tremendous interest in perspectives on music from voices outside academia–for example the second most viewed article was by Douek, entitled “*Music and emotion – a composer's perspective*”—reflections on being a film composer, that is an indication of the widespread popular interest in the topic of music and the brain. Other musicians and artists have joined this deepening interest in the relation of music to perception, emotion, cognition and have welcomed insights from neuroscience (Fleming, 2024). An exciting and growing area in the confluence of the fields of music and neuroscience, and in the clinical and popular arena, is the exploration of the healing potential of music. A recent conference (December 2023) at the National Institutes of Health, presenting research at the interface of sound and health, was devoted to this topic of Music as Medicine (NIH Workshop, 2023).

Our current Research Topic, *Musical Brain II* explores new discoveries and insights into the relationship between music and the brain and includes fascinating contributions on topics ranging from effects of musical training on auditory processing, rhythmic regularity in song and speech, integration of “what” and “when” musical predictions, contextual factors influencing likeability of music, contributions of reward pathways in mediating musical pleasure in naturalistic listening, an evolutionary and neuroscience based comparative analysis of beat perception and synchronization. We briefly introduce each of the seven contributions and hope these exciting articles stimulate new ideas and further research on the Musical Brain.

- Albury et al. “*Context changes judgements of liking and predictability for melodies*”

This paper reveals the importance of musical context in shaping the liking and predictability of short melodies. When we listen to music, we generate predictions about what is likely to happen next. Previous work has shown that predictability, based on long-term, prior knowledge and immediate stimulus features, plays a key role in evoking musical pleasure (Gold et al., 2019; Gold et al., 2023; Vuust et al., 2022). To test whether musical pleasure is also influenced by the musical context in which it is heard, on-line experiments compared human listeners' judgements of two distinct sets of musical stimuli, when they were presented alone or in combination. The results showed that the same musical stimuli could elicit different responses in different circumstances. These findings demonstrated a context-dependent shift of musical predictability and pleasure, indicating that both predictability and reward in music are experienced, not only based on statistical properties of the music, but also influenced relative to the musical context in which it is heard.

- Leite Filho et al. “*Auditory temporal resolution and backward masking in musicians with absolute pitch*”

The paper studied musicians with absolute pitch (AP), the impressive ability to easily name notes without a reference tone. Although the genesis and basis for this ability is unknown, an earlier study (Fujisaki and Kashino, 2005), indicated that pitch identification is more precise for iterated rippled noise than for bandpass noise, suggesting that temporal cues may play a role in AP. This conjecture is consistent with other studies showing that temporal processing is essential for pitch perception. In order to test whether high temporal acuity may contribute to AP by analyzing the temporal fine structure of sound in pitch perception, Filho and colleagues examined temporal resolution in AP musicians by testing their ability to detect small gaps in noise. They found that gap resolution (in which subjects indicate whenever they perceive a short silent interval during a continuous stimulus) was a good predictor of AP pitch naming precision. However, in contrast, the study found no correlation of absolute pitch with results of another test of temporal ability (backward masking—the modification of hearing threshold for a tone, by a subsequent noisy stimulus). Although very exciting, additional research is needed to further resolve the contribution of temporal cues to AP.

- Jiang et al. “*Neural mechanisms of musical structure and tonality and the effect of musicianship*”

This fMRI neuroimaging study explored the multiple cortical and subcortical neural pathways and structures that are activated by processing tonality and musical syntax across different musical genres with varying degrees of tonality—classical, impressionist and atonal music—as well as the role of musical training and experience in such processing. Study participants (musicians and non-musicians) listened to phrases from the different musical genres in the scanner and performed a classification task (assigning musical phrases to the different genres) outside the scanner. One result from this study was evidence that the dorsal stream of auditory processing, including bilateral inferior frontal gyrus and superior temporal gyrus, all play key roles in perception of tonality. This bilateral representation is intriguing in light of previous work that emphasized right hemisphere dominance for tonality perception (Zatorre, 2001). Other results emphasized the importance of a broad network for musical syntactic processing utilizing right hemisphere structures, including right frontotemporal regions and right pars triangularis (in the frontal cortex) and a bilateral cortical-subcortical network including pallidum and cerebellum. This work adds to the exciting literature exploring the broadly distributed, and overlapping neural representations of tonality and syntax in music and language, emergent hemispheric differences in language and music processing, and the effects of musical training. Some recent studies support the presence of separate auditory cortical areas selective for music (Norman-Haignere et al., 2022; Chen et al., 2023), distinct from the cortical network for language processing, while other finer-grained ECoG studies have discovered that sites that encoded pitch and pitch change in music also represented similar properties in speech (McCarty et al., 2023; Sankaran et al., 2024) suggesting that pitch processing pathways are shared in music and speech. These results and others (Leonard et al., 2019) reveal the multidimensional nature of melody encoding in the auditory brain, consisting of music-specific, language-specific and domain-general sound representations, and hence that brain mechanisms for music and language overlap and are intertwined.

- Huang and Yin “*Phylogenic evolution of beat perception and synchronization: a comparative neuroscience perspective*”

This review article explores the evolutionary origin of music, from the perspective that different aspects of music cognition likely evolved independently. The review focuses on rhythm, and the evolution of beat perception and synchronization (BPS), a key element of music and dance behavior in humans, and propose a new integrative neural circuit model of BPS. Their model, based on interesting but controversial recent findings (Cook et al., 2013; Wilson and Cook, 2016; Ito et al., 2021), challenges an existing hypothesis that only vocal learning species are capable of BPS (Patel, 2006) but see Patel (2024) for a response to these studies. The essence of Patel's Vocal Learning and Rhythmic Synchronization Hypothesis (VLRSH) is that only vocal learners like cetaceans, pinnipeds, primates, songbirds, parrots, hummingbirds are capable of BPS, the idea being that neural changes in auditory-motor forebrain circuitry, driven by the evolution of complex vocal learning, laid the neural foundations for the capacity to synchronize movement to the beat of music. In contrast, Huang and Yin advance the possibility that BPS is a common trait in many species, including non-vocal learners, and offer a new integrative neural model based on ancient shared cortical-basal ganglia-thalamic circuitry subserving timing and rhythm perception.

- Yu et al. “*Perceived rhythmic regularity is greater for song than for speech: examining acoustic correlates of rhythmic regularity in speech and song*”

This article analyzes human perception of rhythmic regularity in speech and song, using acoustically matched and unmatched stimuli (spoken and sung utterances). The subjective ratings were analyzed to ascertain whether study participants perceived the presence or absence of an underlying beat, and the ratings were correlated with stimulus features to identify acoustic metrics of rhythmic regularity in both domains. In addition to confirming that overall, music is more likely than speech to induce beat perception, this study identified two key acoustic features (longer stimulus duration and less spectral flux) that predict listener perception of rhythmic regularity. Complementing this study by Yu and colleagues is an excellent, recent overview of the different neural mechanisms underlying speech vs song (Harris et al., 2023).

- Cappotto et al. “‘*What' and ‘when' predictions modulate auditory processing in a mutually congruent manner*”

During listening, our ongoing musical predictions are based on a combination of harmonic, melodic and rhythmic expectancies. This article explores whether the brain integrates these predictions in a mutually congruent manner—whether rhythmic (“beat” timing) “when” predictions interact with “what” predictions of the melodic musical content. A related question was whether these “when” and “what” predictions arise from different neural substrates and if so, how they are functionally coupled. To address these questions, the study recorded EEG to deviants (MMR—mismatch responses) from human subjects who were presented with streams of tones and were instructed to detect tone repetitions. The stimuli were carefully designed tone streams that were independently manipulated in timing or content in two contexts (fast and slow time scales), enabling dissociation of “what” and “when” predictions while keeping other elements of the stimulus stream intact. Behavioral results indicated congruence of “when” and “what” predictions, arising from a shared and distributed neural substrate (based on EEG source reconstruction) that included a wide network of bilateral auditory cortical and right inferior frontal gyrus. Temporal “when” predictions modulated MMR responses to violations of “what” predictions at predicted time points, associated with a decrease in left parietal cortex activity. This suggests that the left parietal cortex may play a key role in integrating “what” and “when” acoustic information as has been previously shown for speech processing (Orpella et al., 2020). These results lead to an integrated model, compared to other studies that have indicated separable brain mechanisms for beat-based and memory-based predictive processing.

- Gold et al. “*Auditory and reward structures reflect the pleasure of musical expectancies during natural listening*”

Over 20 years ago, neuroimaging research revealed that the joy of listening to music engages ancient reward pathways in the Nucleus Accumbens and Ventral Striatum (VS) (Blood and Zatorre, 2001) and in other auditory processing structures including the right superior temporal gyrus (STG) and the anterior prefrontal cortex (Mas-Herrero et al., 2021). Subsequent research has shown that musical complexity, musical surprise and expectation all play key roles in shaping musical listening pleasure. Listeners tend to prefer music in two sweet spots—when it either validates uncertain musical predictions (high uncertainty and low surprise) or when it violates highly confident predictions (low uncertainty and high surprise). This experimental human behavioral and fMRI study (Gold et al.) tested the hypothesis that pleasure and reward value (and accompanying VS activation) increase when learning about musical structure during natural listening to short musical excerpts. These excerpts were carefully chosen, including a broad musical range from different genres and time periods, that varied widely in surprise and uncertainty. The results, correlating subject liking ratings and BOLD neuroimaging, confirmed the hypothesis that average liking correlates with VS activity and also suggested that the VS conveys reward signals based on musical expectations. This strengthens the evidence for the link between musical expectancies and to pleasure and its neural substrates (in the VS and STG). These insights provide a foundation for future studies of the role of other variables, such as the influence of prior musical knowledge and experience, and temporal dynamics, developing a finer-grain moment-to-moment analysis of unfolding musical surprise, prediction and pleasure during listening.

Conclusion: In summary, this new Special Issue, continues the Research Topic of the Musical Brain, and brings together many strands of research that deepen our understanding of a wide range of topics, including the evolution of beat perception, the psychology and neuroscience of rhythmic and melodic musical perception, prediction and pleasure, the relation of music and spoken language, and the perceptual and brain changes with years of musical training. This exciting field, at the fertile confluence of music and neuroscience, continues to grow and inspire musicians, healthcare providers, art therapists, educators as well as research scientists.

## Author contributions

JF: Writing – original draft, Writing – review & editing. AB: Writing – review & editing. JG: Writing – review & editing. JI: Writing – review & editing. IP: Writing – review & editing. RZ: Writing – review & editing.
